# Zero-Fluoroscopy Pulsed Field Ablation with a Variable Loop Circular Catheter in a Patient with a Permanent Pacemaker

**DOI:** 10.1016/j.cjco.2025.11.004

**Published:** 2025-11-17

**Authors:** William K. Chan, Umjeet S. Jolly

**Affiliations:** a Heart Rhythm Program, Waterloo Regional Health Network, Kitchener, Ontario, Canada; b Department of Medicine, McMaster University, Hamilton, Ontario, Canada

**Keywords:** catheter ablation, atrial fibrillation, zero fluoroscopy, cardiac implantable electronic devices, pulsed field ablation

**Only a paucity of data is available on the use of a variable loop circular catheter (VLCC) for pulsed field ablation (PFA) in patients with cardiac implantable electronic devices (CIEDs). To date, this case report is the first to demonstrate fluoro-less pulmonary vein isolation using a VLCC in a patient with a dual-chamber, permanent pacemaker**.

## Case

A 76-year-old woman with tachy-brady syndrome underwent implantation of a dual-chamber permanent pacemaker 2 years earlier. Her pacemaker was programmed to AAI-DDD mode (automatic switching mode to minimize ventricular pacing), with a lower rate of 60 beats per minute (bpm), requiring 64% atrial pacing. Despite receiving bisoprolol 2.5 mg once daily, she presented with recurrent symptomatic paroxysmal atrial fibrillation and felt more fatigued with higher doses of bisoprolol. She had normal left ventricular ejection fraction and left atrial size. After an informed discussion about rhythm control options, she ultimately decided to pursue catheter ablation for definitive therapy.

During the day of the procedure, the patient’s underlying rhythm was sinus bradycardia at 50 bpm, and her pacemaker was programmed to VVI 40 bpm in anticipation of any vagal responses from the PFA.

**Figure 1:**
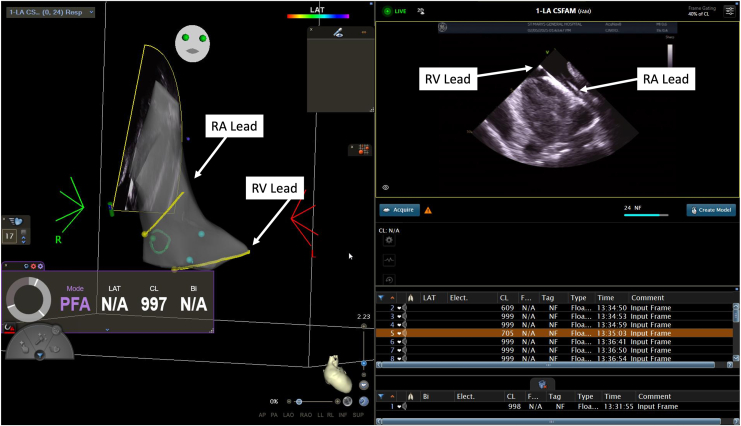
Intracardiac echocardiography (ICE) visualization of intracardiac pacemaker leads: ICE catheter in the right atrium with a posterior tilt and right steer to visualize 2 intracardiac pacemaker leads in the superior vena cava. **Upper yellow line** indicates right atrial (RA) lead; **bottom yellow line** indicates right ventricular (RV) lead; **green circle** indicates coronary sinus os; **light blue dots** indicate tricuspid annulus. Legend: upper right yellow line = right atrial (RA) lead, bottom right yellow line – right ventricular (RV) lead, green circle – coronary sinus os, light blue dots – tricuspid annulus

Under general anesthesia, 2 right-sided femoral venous accesses were obtained under direct ultrasound guidance. An intracardiac echocardiography (ICE) catheter (SOUNDSTAR, Biosense Webster, Irvine, CA) was inserted into the right atrium. The right atrial and ventricular lead positions were demarcated by ICE and projected onto the 3-dimensional (3-D) CARTO map (Biosense Webster; Fig. 1). Detailed 3-D geometry of the left atrium, using the CARTOSOUND Fast Anatomical Mapping (FAM) module, (Biosense Webster) was created, powered by artificial intelligence. No FAM was required for the right atrium, reducing the risk of lead dislodgement.

To achieve transseptal access, a long J-wire was inserted into the superior vena cava and visualized with ICE. A VIZIGO sheath (Biosense Webster) was advanced with the dilator over the wire. The wire was removed, and a transseptal needle was advanced through the sheath but not beyond the dilator. The entire apparatus was carefully dragged caudally until the tip of the dilator fell into the fossa ovalis under direct ICE guidance to confirm that no leads were entangled (Video 1, view video online). The needle was advanced through the dilator with penetration through the fossa in an anterior-inferior orientation toward the left superior pulmonary vein. Once the wire was in the pulmonary vein, the septum was dilated, and the sheath was advanced into the left atrium. The VLCC (VARIPULSE, Biosense Webster) was introduced through this sheath without need for further catheter exchanges to minimize risk of thromboembolism. Our technique was described previously, with the VLCC used as the FAM, mapping, and ablation catheter all in one.^1^ Pulmonary vein isolation (PVI) was performed according to the standard workflow recommendations of 4 ablations per pulmonary vein. All pulmonary veins were acutely isolated, showing entrance block. In total, 24 ablations were delivered (72 applications) with a left atrial dwell time of 44 minutes, and a total skin-to-skin time of 75 minutes. Total fluoroscopy time was zero.

Pacemaker interrogation at the end of the procedure revealed unchanged and normal values for sensing, pacing, and impedance of both leads. No programming changes were made. No acute complications occurred, and the patient was discharged on the same day. Follow-up evaluation at 3 months showed no recurrence of atrial fibrillation on pacemaker interrogation, improvement of patient symptoms, and stable pacemaker parameters.

## Discussion

Herein, we describe the first case report of using a VLCC to perform PFA on a patient with a CIED using a simplified fluoro-less workflow. Only a paucity of data is available on the safety of using the VLCC on patients with CIEDs, although evidence exists for other bipolar PFA catheters, namely the pentaspline catheter.^2^ In theory, the strong electrical field generated by PFA can cause damage to CIED electronic components, including pacing inhibition, inappropriate defibrillator therapies, and/or mode switches. This issue is of particular concern in patients who are pacing-dependent and have more-complex devices/treatments, such as defibrillators and cardiac resynchronization therapy. However, each PFA catheter system is unique in its design and requires individual safety validation in those with CIEDs. Unlike the pentaspline catheter, the VLCC delivers fewer pulse trains (3 vs 5), separated by a longer pause between each train (10 s vs 300 ms), which could theoretically reduce the duration of pacing inhibition. One case report demonstrated effective PVI ablation with no adverse effects despite the VLCC coming as close as 43 mm to the tip of the right atrial lead using computed tomography reconstruction.^3^ Although our case supports the continued safety of using a VLCC in a patient with a pacemaker, longer-term follow-up is required to confirm both arrhythmia-free survival and stable long-term device performance.

Fluoro-less procedures have been reported previously in patients with CIED pertaining to the use of focal radiofrequency ablation, a 3-D mapping system, and ICE.^4^ None exist for PFA. In our case, ICE allowed each lead position to be projected on the 3-D map in real time. Specialized maneuvers can minimize lead dislodgement, such as a direct approach for transseptal access into the fossa ovalis and avoiding 180-degree manipulation of catheters in the right atrium.^4^ The direct approach requires a separate navigational catheter through the deflectable sheath to landmark the fossa. The subsequent introduction of needle and dilator may displace the desired puncture location. An ICE-guided dropdown of the transeptal needle from the superior vena cava requires careful attention to avoid lead entanglement, but it will sit more naturally in the fossa once the dropdown is complete. By not having additional catheters in the right atrium and avoiding further unnecessary matrix generation, our procedure was thereby streamlined and may be more appealing to operators who wish to perform fluoro-free procedures.

Ultimately, dedication and perseverance are required to perform fluoro-less ablation in patients with CIEDs, as the operator learning curve is steep (>30 cases), case times are lengthier, and the level of incentive to transition from low-fluoroscopy procedures is low. Fluoro-less procedures should always be balanced against the potential for lead-related complications, especially for higher-risk patients, such as those with recent implants, biventricular devices with coronary sinus leads, and preexisting lead-related issues. Fortunately, such occurrences are rare.^5^ Payoffs accompany performing fluoro-free ablation, including elimination of radiation exposure, reduction of orthopedic issues, and achievement of cost-savings on a fixed C-arm. The use of both ICE and electroanatomic mapping also may be associated with higher durable PVI compared to fluoroscopy only, and is better integrated under one mapping/ablating system.

**Novel Teaching Points**

• Fluoroscopy-free procedures using a VLCC in patients with a CIED require careful planning and manipulation, but they can be safe and feasible, as described in this index case.
• The presence of a CIED should not necessarily dissuade operators from offering atrial fibrillation catheter ablation using PFA with a zero-fluoroscopy, simplified workflow.

## Supplementary Material

Video 1 Dropdown of transeptal needle from the superior vena cava (SVC) to the fossa ovalis Real-time visualization of the dropdown of the sheath from the SVC to the fossa ovalis. Emergence of the transeptal needle at the desired puncture location indicated by tenting at the fossa ovalis. Counterclockwise and clockwise pan of the ICE catheter to confirm no lead entanglement. Slight counterclockwise turn of the sheath to direct the needle anteriorly across the septum towards the left atrial appendage.

## References

[1] Chan W.K., Jolly U.S. (2025). A simplified, fluoroless workflow for atrial fibrillation pulsed field electroporation. J Arrhythm.

[2] Iacopino S., Tondo C., Bianchi S. Safety and feasibility of pulsed field ablation with a pentaspline catheter in patients with cardiac implantable electronic devices: a multicentre experience.

[3] Iqbal S.U.R., Kueffer T., Panakal A., Roten L., Reichlin T. (2025). Safety of pulsed field ablation using a variable loop circular catheter in a patient with a cardiac implantable electronic device. Hear Case Rep.

[4] Shimamoto K., Yamagata K., Wakamiya A. (2022). Zero-fluoroscopy ablation in patients with cardiac electronic implantable devices. J Cardiovasc Electrophysiol.

[5] Iqbal A.M., Li K.Y., Mahmood M., Gautam S. (2023). Safety of fluoroless radiofrequency catheter ablation for atrial fibrillation in patients with pre-existing cardiac implantable electronic device: a single-center study. Pacing Clin Electrophysiol.

